# Author Correction: Immunogenicity and efficacy of CNA25 as a potential whole-cell vaccine against systemic candidiasis

**DOI:** 10.1038/s44321-025-00263-x

**Published:** 2025-12-12

**Authors:** Satya Ranjan Sahu, Abinash Dutta, Doureradjou Peroumal, Premlata Kumari, Bhabasha Gyanadeep Utakalaja, Shraddheya Kumar Patel, Narottam Acharya

**Affiliations:** 1 Department of Infectious Disease Biology, Institute of Life Sciences, Bhubaneswar, Odisha 751023 India; 2 Regional Center for Biotechnology, Faridabad, Haryana 751021 India

## Abstract

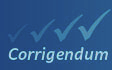

**Correction to:** *EMBO Molecular Medicine* (2024) 16:1254–1283. 10.1038/s44321-024-00080-8 | Published online 23 May 2024

**Figure 9 is withdrawn and replaced**.

**Figure 9:**
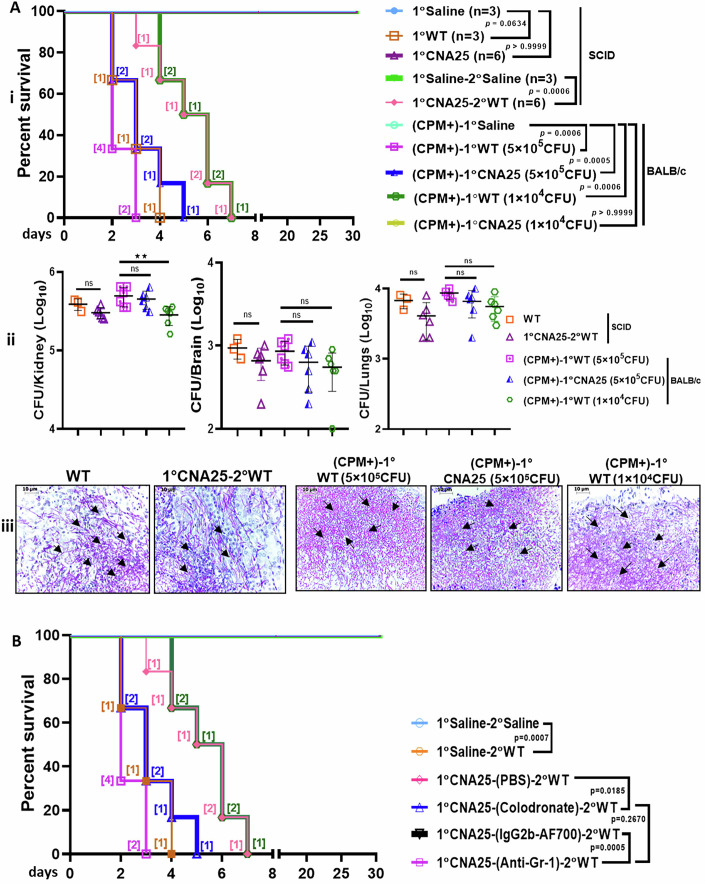
**Original.**

**Figure 9:**
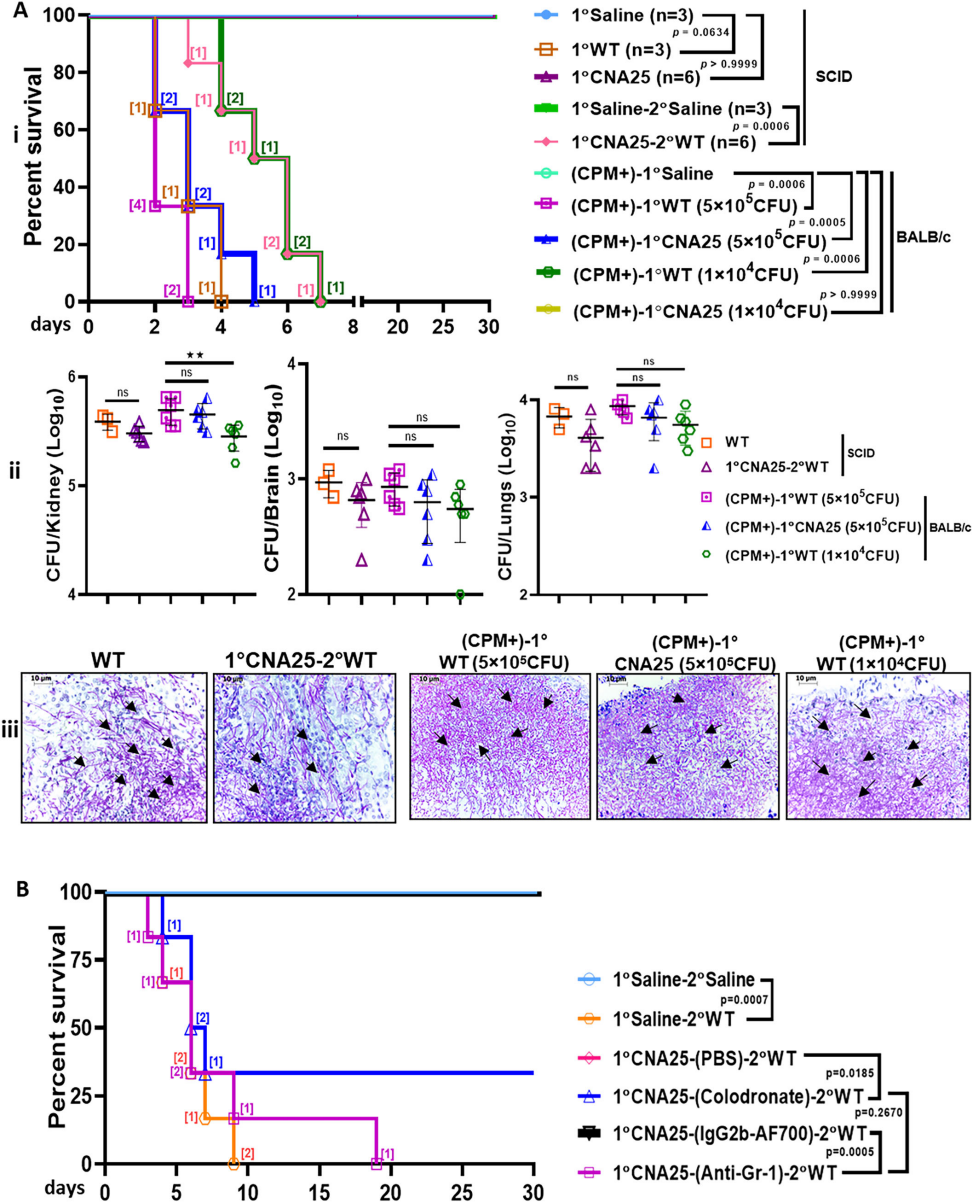
**Corrected.**

**Table EV1 is withdrawn and replaced**.

**Original published Table EV1**

**Table EV1:** Survival rate of mice challenged with *Candida* strains.

Strains	Mean death time (days)
***C. albicans*** ***and non-albicans sp. virulence***	
*C. albicans*	6
*C. parapsilosis*	20
*C. tropicalis*	9
*C. glabrata*	9
**Cross-species protection**	
(1°-IV) Saline-(2°) *C. albicans*	7
(1°-IV) CNA25 HK-(2°) *C. albicans*	5
(1°-IV) CNA25-(2°) *C. tropicalis*	19
**Differential mode of immunization**	
(1°-Oral) CNA25-(2°) WT	22
(1°-Oral) Saline-(2°) WT	7
(1°-IP) Saline-(2°) WT	5
(1°-IP) CNA25-(2°) WT	10
(1°-SC) Saline-(2°) WT	5
(1°-SC) CNA25-(2°) WT	7
**Dectin-1 and TLR2 depletion**	
1°Saline-2°WT	6
1°CNA25-(Anti-Dectin-1)-2°WT	9
1°CNA25-(Anti-TLR2)-2°WT	undefined
**CD4 and CD8 depletion**	
1°Saline-2°WT	6
1°CNA25-(Anti-CD4)-2°WT	7
1°CNA25-(Anti CD8)-2°WT	10
**IFNγ, IL17 and TNFα depletion**	
1°Saline-2°WT	6
1°CNA25-(Anit-IFNγ)-2°WT	6
1°CNA25-(Anti-IL17)-2°WT	6
1°CNA25-(Anti-TNFα)-2°WT	8
**SCID mice**	
1°WT (n = 3)	2
1°CNA25-2°WT (n = 6)	5
**Cyclophosphamide treatment**	
(CPM+)-1°WT (5 × 10^5^CFU)	2
(CPM+)-1°CNA25 (5 × 10^5^CFU)	3
(CPM+)-1°WT (1 × 10^4^CFU)	6

**Corrected published Table EV1**

**Table EV1:** Survival rate of mice challenged with *Candida* strains.

Strains	Median death time (days)
***C. albicans*** ***and non-albicans sp. virulence***	
*C. albicans*	6
*C. parapsilosis*	20
*C. tropicalis*	9
*C. glabrata*	9
**Cross-species protection**	
(1°-IV) Saline-(2°) *C. albicans*	7
(1°-IV) CNA25 HK-(2°) *C. albicans*	5
(1°-IV) CNA25-(2°) *C. tropicalis*	19
**Differential mode of immunization**	
(1°-Oral) CNA25-(2°) WT	22
(1°-Oral) Saline-(2°) WT	7
(1°-IP) Saline-(2°) WT	5
(1°-IP) CNA25-(2°) WT	10
(1°-SC) Saline-(2°) WT	5
(1°-SC) CNA25-(2°) WT	7
**Dectin-1 and TLR2 depletion**	
1°Saline-2°WT	6
1°CNA25-(Anti-Dectin-1)-2°WT	9
1°CNA25-(Anti-TLR2)-2°WT	undefined
**CD4 and CD8 depletion**	
1°Saline-2°WT	6
1°CNA25-(Anti-CD4)-2°WT	7
1°CNA25-(Anti CD8)-2°WT	10
**IFNγ, IL17 and TNFα depletion**	
1°Saline-2°WT	6
1°CNA25-(Anit-IFNγ)-2°WT	6
1°CNA25-(Anti-IL17)-2°WT	6
1°CNA25-(Anti-TNFα)-2°WT	8
**SCID mice**	
1°WT (n = 3)	2
1°CNA25-2°WT (n = 6)	5
**Cyclophosphamide treatment**	
(CPM+)-1°WT (5 × 10^5^CFU)	2
(CPM+)-1°CNA25 (5 × 10^5^CFU)	3
(CPM+)-1°WT (1 × 10^4^CFU)	6

The authors contacted the journal after identifying errors in Figure 9 and Table EV1 of the published article. The authors provided the journal with the corrected figure and table, and after review, the journal withdraws and replaces Figure 9 and Table EV1.

Author statement:

In Figures 9A and 9B, we found that the graphs were inadvertently duplicated with different graph legends. However, the published source data and their descriptions in the results section remain correct. The corrected Figure 9, now provided, maintains all original conclusions.

Additionally, a typographical correction in Table EV1, correcting ‘mean death time’ to ‘median death time,’ does not affect the study’s findings.

All authors agree to this correction and apologise for any inconvenience caused.

