# Supplementary material for: Immunogenicity and efficacy of CNA25 as a potential whole-cell vaccine against systemic candidiasis
Source: EMBO Mol Med. 2024 May 23;16(6):4. doi: 10.1038/s44321-024-00080-8 (PMC11178797; doi:10.1038/s44321-024-00080-8)
Supplement: Supplementary file 1 — Table EV1 [file 44321_2024_80_MOESM1_ESM.docx]

**Table- EV1: Survival rate of mice challenged with *Candida* strains**

| **Strains** | **Mean death Time(days)** |
| --- | --- |
| ***C. albicans* and non-albicans sp. virulence** | |
| *C. albicans* | 6 |
| *C. parapsilosis* | 20 |
| *C. tropicalis* | 9 |
| *C. glabrata* | 9 |
| **Cross-species protection** | |
| (1°-IV) Saline-(2°) *C. albicans* | 7 |
| (1°-IV) CNA25 HK-(2°) *C. albicans* | 5 |
| (1°-IV) CNA25-(2°) *C. tropicalis* | 19 |
| **Differential mode of immunization** | |
| (1°-Oral) CNA25-(2°) WT | 22 |
| (1°-Oral) Saline-(2°) WT | 7 |
| (1°-IP) Saline-(2°) WT | 5 |
| (1°-IP) CNA25-(2°) WT | 10 |
| (1°-SC) Saline-(2°) WT | 5 |
| (1°-SC) CNA25-(2°) WT | 7 |
| **Dectin-1 and TLR2 depletion** | |
| 1°Saline-2°WT | 6 |
| 1°CNA25-(Anti-Dectin-1)-2°WT | 9 |
| 1°CNA25-(Anti-TLR2)-2°WT | undefined |
| **CD4 and CD8 depletion** | |
| 1°Saline-2°WT | 6 |
| 1°CNA25-(Anti-CD4)-2°WT | 7 |
| 1°CNA25-(Anti CD8)-2°WT | 10 |
| **IFNγ, IL17 and TNFα depletion** | |
| 1°Saline-2°WT | 6 |
| 1°CNA25-(Anit-IFNγ)-2°WT | 6 |
| 1°CNA25-(Anti-IL17)-2°WT | 6 |
| 1°CNA25-(Anti-TNFα)-2°WT | 8 |
| **SCID mice** | |
| 1°WT (n=3) | 2 |
| 1°CNA25-2°WT (n=6) | 5 |
| **Cyclophosphamide treatment** | |
| (CPM+)-1°WT (5×10^5^CFU) | 2 |
| (CPM+)-1°CNA25 (5×10^5^CFU) | 3 |
| (CPM+)-1°WT (1×10^4^CFU) | 6 |
