# Supplementary material for: Immunogenicity and efficacy of CNA25 as a potential whole-cell vaccine against systemic candidiasis
Source: EMBO Mol Med. 2024 May 23;16(6):4. doi: 10.1038/s44321-024-00080-8 (PMC11178797; doi:10.1038/s44321-024-00080-8)
Supplement: Supplementary file 2 — Table EV2 [file 44321_2024_80_MOESM2_ESM.docx]

**Table- EV2: Different statistical parameters obtained from regression analyses with respect to fungal strains and blood cell counts**

| **Parameters** | **WT** | **CNA25** | **1°CNA25-2°WT** |
| --- | --- | --- | --- |
| **WBCs** | | | |
| R^2^ | 0.8044 | 0.2531 | 0.06881 |
| Equation | Y = 0.6863*X + 6.144 | Y = -0.08491*X + 7.595 | Y = -0.04277*X + 7.555 |
| *p* value | <0.0001 | 0.0033 | 0.154 |
| **Granulocytes** | | | |
| R^2^ | 0.4936 | 0.2199 | 0.03214 |
| Equation | Y = 0.4157*X + 1.943 | Y = -0.05717*X + 2.955 | Y = -0.01228*X + 2.620 |
| P value | 0.0017 | 0.0078 | 0.3262 |
| **Monocytes** | | | |
| R^2^ | 0.5656 | 0.2051 | 0.03315 |
| Equation | Y = 0.1001*X + 0.3908 | Y = -0.01144*X + 0.6336 | Y = -0.002297*X + 0.3998 |
| *p* value | 0.0005 | 0.0155 | 0.3186 |
| **Lymphocytes** | | | |
| R^2^ | 0.3999 | 0.5307 | 0.5051 |
| Equation | Y = -0.2005*X + 3.914 | Y = -0.1107*X + 5.956 | Y = -0.08524*X + 6.522 |
| *p* value | 0.0064 | <0.0001 | <0.0001 |
| **Platelets** | | | |
| R^2^ | 0.7555 | 0.4319 | 0.1802 |
| Equation | Y = 45.66*X + 664.0 | Y = -7.376*X + 801.4 | Y = -6.805*X + 847.4 |
| *p* value | <0.0001 | <0.0001 | 0.0155 |
