# Supplementary material for: Immunogenicity and efficacy of CNA25 as a potential whole-cell vaccine against systemic candidiasis
Source: EMBO Mol Med. 2024 May 23;16(6):4. doi: 10.1038/s44321-024-00080-8 (PMC11178797; doi:10.1038/s44321-024-00080-8)
Supplement: Supplementary file 3 — Appendix [file 44321_2024_80_MOESM3_ESM.pdf]

Appendix Information

Immunogenicity and efficacy of CNA25, a potential live-attenuated vaccine strain against hematogenously disseminated candidiasis

Satya Ranjan Sahu[1,2#], Abinash Dutta[1,#], Doureradjou Peroumal[1], Premlata Kumari[1,2], Bhabasha Gyanadeep Utakalaja[1,2], Shraddheya Patel[1,2], and Narottam Acharya[1,*]

[1]Department of Infectious Disease Biology, Institute of Life Sciences, Bhubaneswar-751023, India.

[2]Regional center for Biotechnology, Faridabad-751021, India.

# contributed equally.

*Correspondence to:
Narottam Acharya, Phone: 91-674-2304278, Fax: 91-674-230
0728 E-mail: narottam_acharya@ils.res.in;
narottam74@gmail.com

Running title: CNA25 as antifungal vaccine

Table of Content **Page**

Table of Content	Page
1. Appendix Figure S1	3
2. Appendix Figure S2	4
3. Appendix Figure S3	5
4. Appendix Figure S4	6

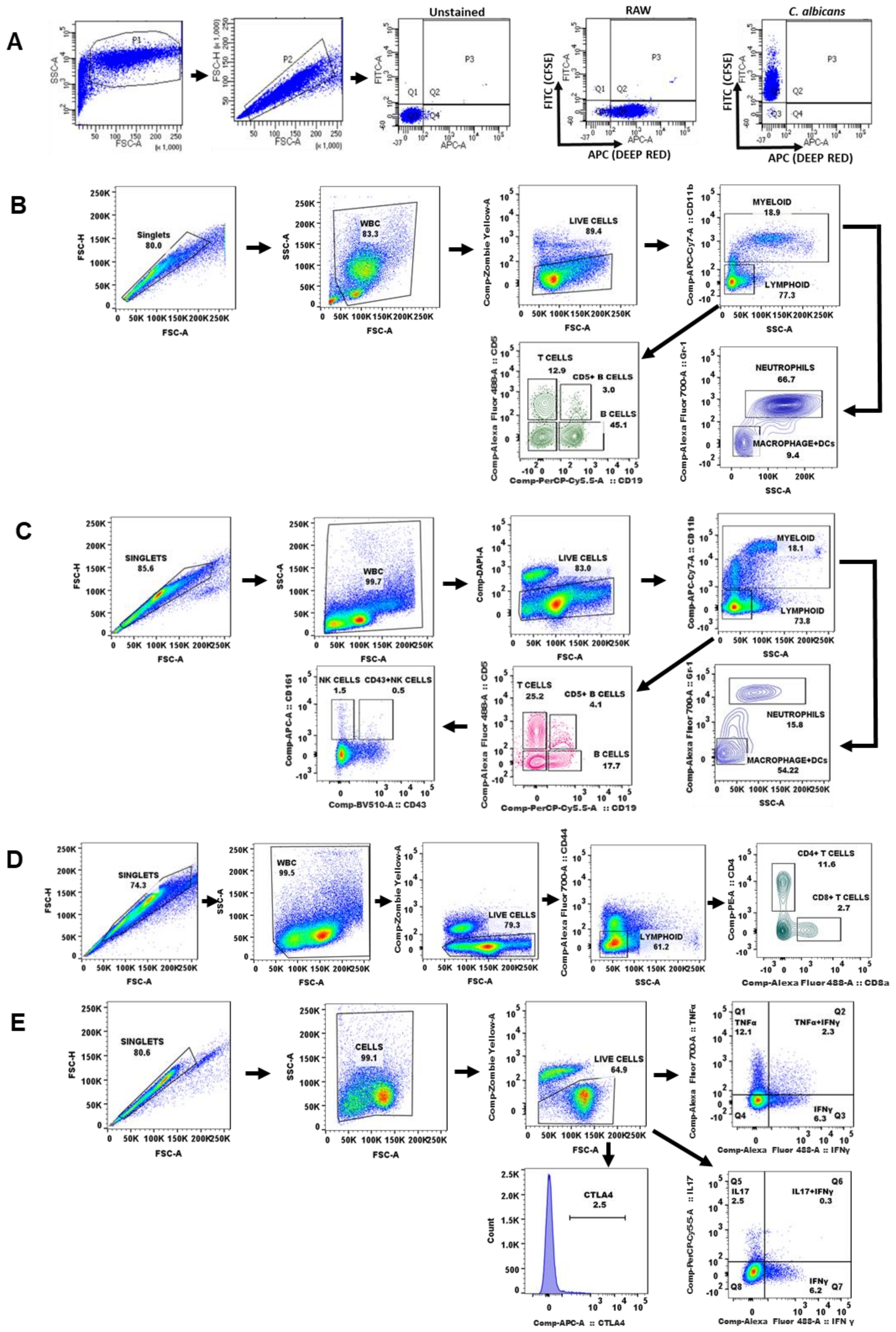

Appendix Figure S1. (A) Gating strategy for Macrophage-fungal cells interaction assay along with single cells control: Debris was eliminated by SSC-A vs FSC-A followed by singlet discrimination by FSC- H vs FSC-A gating then *C. albicans* - Macrophage double positive population was determined by FITC (CFSE) vs APC (Deep reed). Cells were either unstained or stained with CFSE or Deep red dye and gated. (**B)** Gating strategy of Circulating Immune cells: Singlet cells were selected based on FSC- H vs FSC-A, then debris was eliminated by SSC-A vs FSC-A followed by live-dead discrimination using Zombie Yellow-A vs FSC-A gating. Live cells were further differentiated into Lymphoid and Myeloid populations by using APC-Cy7-A::CD11b vs SSC-A; Lymphoid cells were further discriminated into T, B, and $CD5^+$ B (B1) cells by Alexa Fluor 488-A::CD5 vs PerCP-Cy5-5-A::CD19, whereas myeloid cells were discriminated into neutrophils and Macrophage + DCs by Alexa Fluor 700-A::Gr-1 vs SSC-A. (**C)** Gating strategy of Splenic Immune cells: Singlet cells were selected based on FSC-H vs FSC-A, then debris was eliminated by SSC-A vs FSC-A followed by live-dead discrimination using DAPI-A vs FSC-A plot. Live cells were further differentiated into Lymphoid and Myeloid populations by using APC-Cy7-A::CD11b vs SSC-A; Lymphoid cells were further discriminated into T, B, and B1 cells by Alexa Fluor 488-A::CD5 vs PerCP-Cy5-5-A::CD19 whereas myeloid cells were discriminated into neutrophils and Macrophage + DCs by Alexa Fluor 700-A::Gr-1 vs SSC-A. The double negative ($CD5^-$ and $CD19^-$) Lymphoid sub-population further differentiated into NK cells and $CD43^+$ NK cells by using a plot of APC-A::CD161 vs BV510-A::CD43. (**D)** Gating strategy of Splenic T cell subsets: Singlet cells were selected based on FSC-H vs FSC-A, then debris was eliminated by SSC-A vs FSC-A followed by live-dead discrimination using Zombie Yellow-A vs FSC-A gating. From the live cells Lymphoid population was selected by using Alexa Fluor 700-A::CD44 Vs SSC-A and Lymphoid cells were further discriminated into $CD4^+$ T cells and $CD8^+$ T cells by using a plot of PE-A::CD4 vs Alexa Fluor 488-A :: CD8a. (**E)** Gating strategy of Splenic $CD4^+$ T cells subpopulation: Singlet cells were selected based on FSC-H vs FSC-A, then debris was eliminated by SSC-A vs FSC-A followed by live-dead discrimination using Zombie Yellow-A Vs FSC-A gating. The live cells were differentiated into three sets of population; TNFα and IFNγ producing $CD4^+$ T cells were discriminated by Alexa Fluor 700 :: TNFα vs Alexa Fluor 488-A::IFNγ, IL17 producing $CD4^+$T cells were determined using PerCP-Cy5-5-A::IL17 vs Alexa Fluor 488-A::IFNγ and CTLA4 was determined by histogram using APC-A:: CTLA4. Some of the representative bivariate plots were taken from the original data provided in Fig. EV2, Fig. EV4, and appendix Fig. S3,

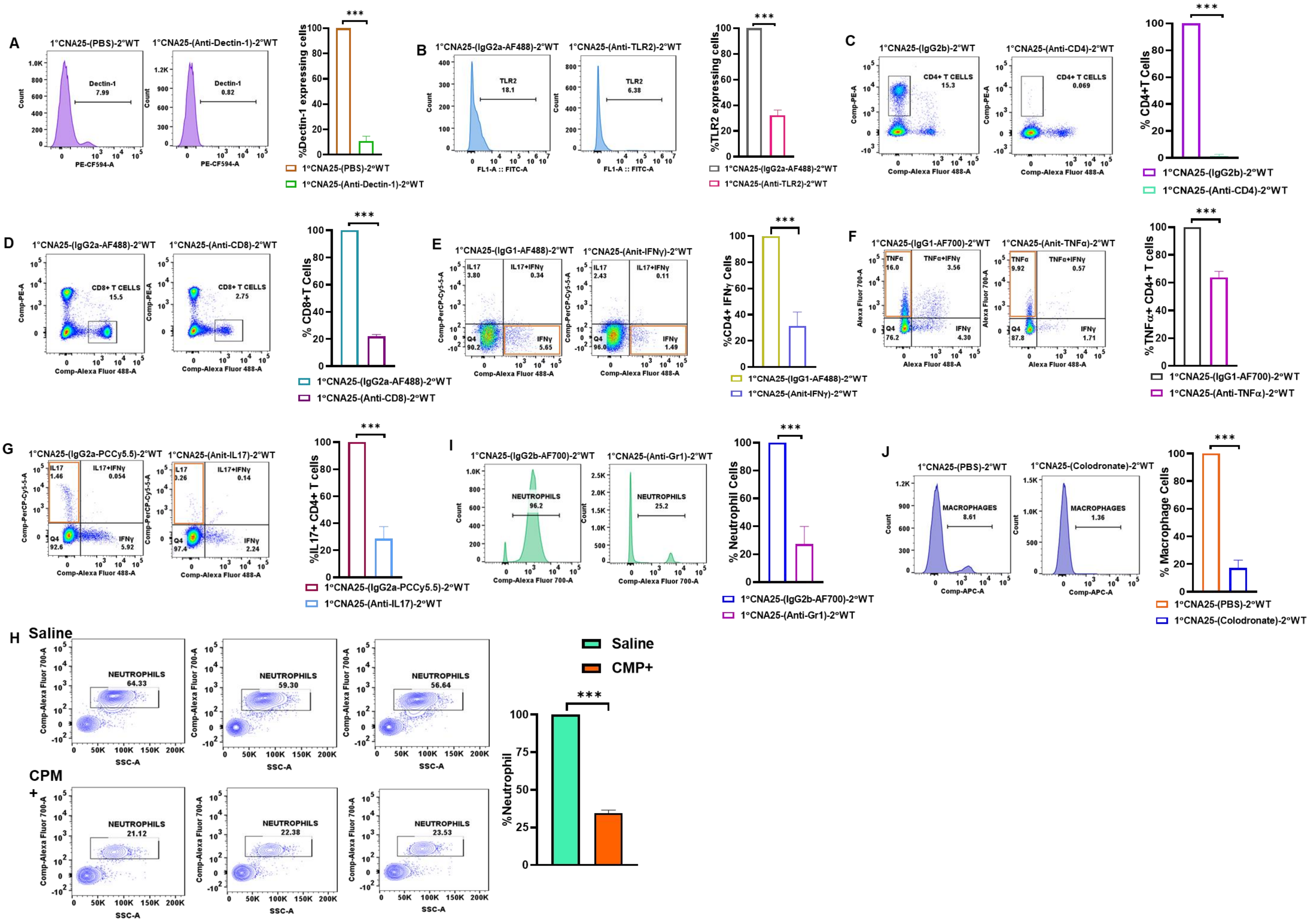

Appendix Figure S2. (**A**) A representative histogram plot showing the percent Dectin-1 positive cells in the blood of mice groups injected with anti-Dectin-1 antibody and respective control (1°CNA25-(Anti-Dectin-1)-2°WT and 1°CNA25-(PBS)-2°WT) group having cell count on the y-axis and PE-CF594-A on the x-axis and estimated as shown in the bar graph. (**B**) A representative histogram plot showing the percent positive of TLR2 expressing cells in the blood of TLR2 depleted (1°CNA25-(Anti-TLR2)-2°WT) and isotype control (1°CNA25-(IgG2a-AF488)-2°WT) group having cell count on the y-axis and FITC-A on the x-axis and estimated as shown in the bar graph. (**C**) A representative bivariate pseudo plot showing the percent positive CD4+ T cells in spleen of CD4 depleted (1°CNA25-(Anti-CD4)-2°WT) and isotype control (1°CNA25-(IgG2b)-2°WT) group having PE conjugated CD4 on y-axis and Alexa fluor 488 conjugated CD8 on x-axis and estimated as shown in the bar graph. (**D**) A representative bivariate pseudo plot showing the percent positive CD8+ T cells in splenocytes of CD8 depleted (1°CNA25-(Anti-CD8)-2°WT) and isotype control (1°CNA25-(IgG2a)-2°WT) group having PE conjugated CD4 on y-axis and Alexa fluor 488 conjugated CD8 on x-axis and estimated as shown in the bar graph. (**E**) A representative bivariate pseudo plot showing the percent positive IFNγ+ CD4+ T cells in splenocytes of IFNγ+ -depleted (1°CNA25-(Anit-IFNγ)-2°WT) and isotype control (1°CNA25-(IgG1-AF488)-2°WT) group having PerCP Cy5.5 conjugated IL17 on y-axis and Alexa fluor 488 conjugated IFNγ on x-axis and estimated as shown in the bar graph. (**F**) A representative bivariate pseudo plot showing the percent positive TNFα+ CD4+ T cells in splenocytes of TNFα+-depleted (1°CNA25-(Anti-TNFα)-2°WT) and isotype control (1°CNA25-(IgG1-AF700)-2°WT) group having Alexa Fluor 700 conjugated TNFα on y-axis and Alexa fluor 488 conjugated IFNγ on x-axis and estimated as shown in the bar graph. G. A representative bivariate pseudo plot showing the percent positive IL17+ CD4+ T cells in splenocytes of IL17+-depleted (1°CNA25-(Anti-IL17)-2°WT) and isotype control (1°CNA25-(IgG2a-PCCy 5.5)-2°WT) having PerCP Cy5.5 conjugated IL17 on y-axis and Alexa fluor 488 conjugated IFNγ on x-axis and estimated as shown in the bar graph. (**H**) A representative bivariate contour plot having Alexa Fluor 700 conjugated Gr-1 Ly-6G/Ly- 6C on the y-axis and Side scatter on the x-axis for the determination of neutrophil percent populations in blood circulation of saline and Cyclophosphamide (200 mg/Kg) treated mice using Flowjo v8.0.2 Software and estimated as shown in the bar graph. Statistical significance in the above graphs was determined using an unpaired t-test. **** $p \leq 0.0001$; *** $p \leq 0.001$; ** $p \leq 0.01$; * $p \leq 0.05$. (**I**) A representative histogram plot showing the percent positive of neutrophil cells in blood of neutrophil depleted (1°CNA25-(Anti-Gr1)-2°WT) and isotype control (1°CNA25-(IgG2b-AF700)-2°WT) group having cell count on y-axis and Gr1 conjugated Alexa Fluor700-A on x-axis and estimated as shown in the bar graph. (**J**) A representative histogram plot showing the percent positive of Macrophage cells in the blood of macrophage depleted (1°CNA25-(clodronate)-2°WT) and control (1°CNA25-(PBS)-2°WT) group having cell count on the y-axis and APC conjugated F4/80 on the x-axis. Bar graph showing the percent reduction of macrophage-depleted cells upon clodronate treatment as compared to the PBS control group.

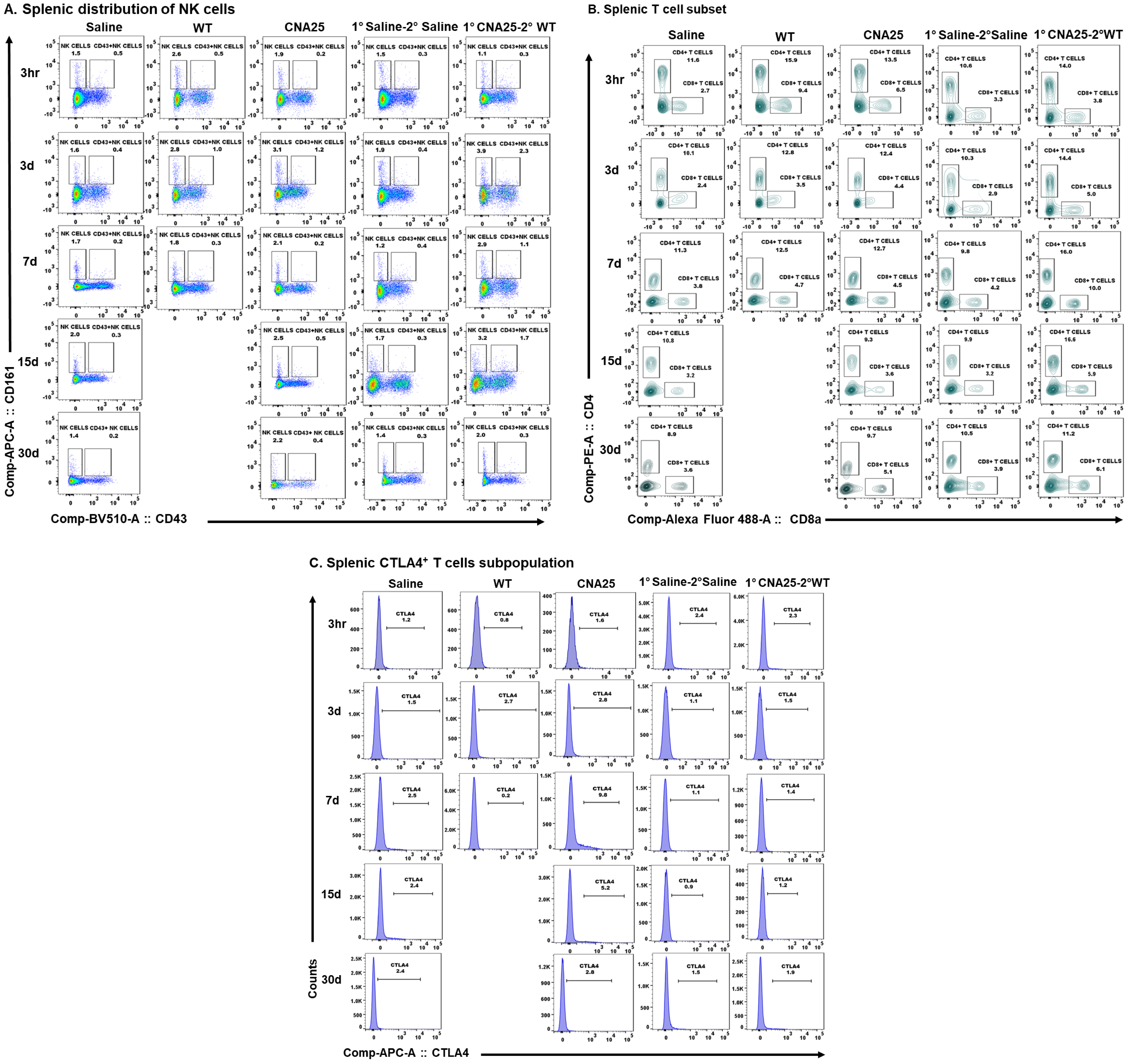

Appendix Figure S3. (**A**) Representative bivariate contour plots of each time point having APC conjugated CD161 on the y-axis and BV510 conjugated CD43 on the x-axis for the analysis of the compartmental distribution of splenic NK cells and CD43+ NK cells using Flowjo v8.0.2 Software are shown. (**B**) Representative bivariate contour plots of all time points having PE conjugated CD4 on the y-axis and Alexa fluor 488 conjugated CD8a on the x-axis for the analysis of the compartmental distribution of splenic T cells subpopulation (CD4+ and CD8+ T cells) using Flowjo v 8.0.2 Software are shown. (**C**) Representative Histograms of all time points having cell count on the y-axis and APC conjugated CTLA4 on the x-axis for the determination of percent positive CTLA4+ CD4+ T cells using Flowjo v8.2.0 Software are given.

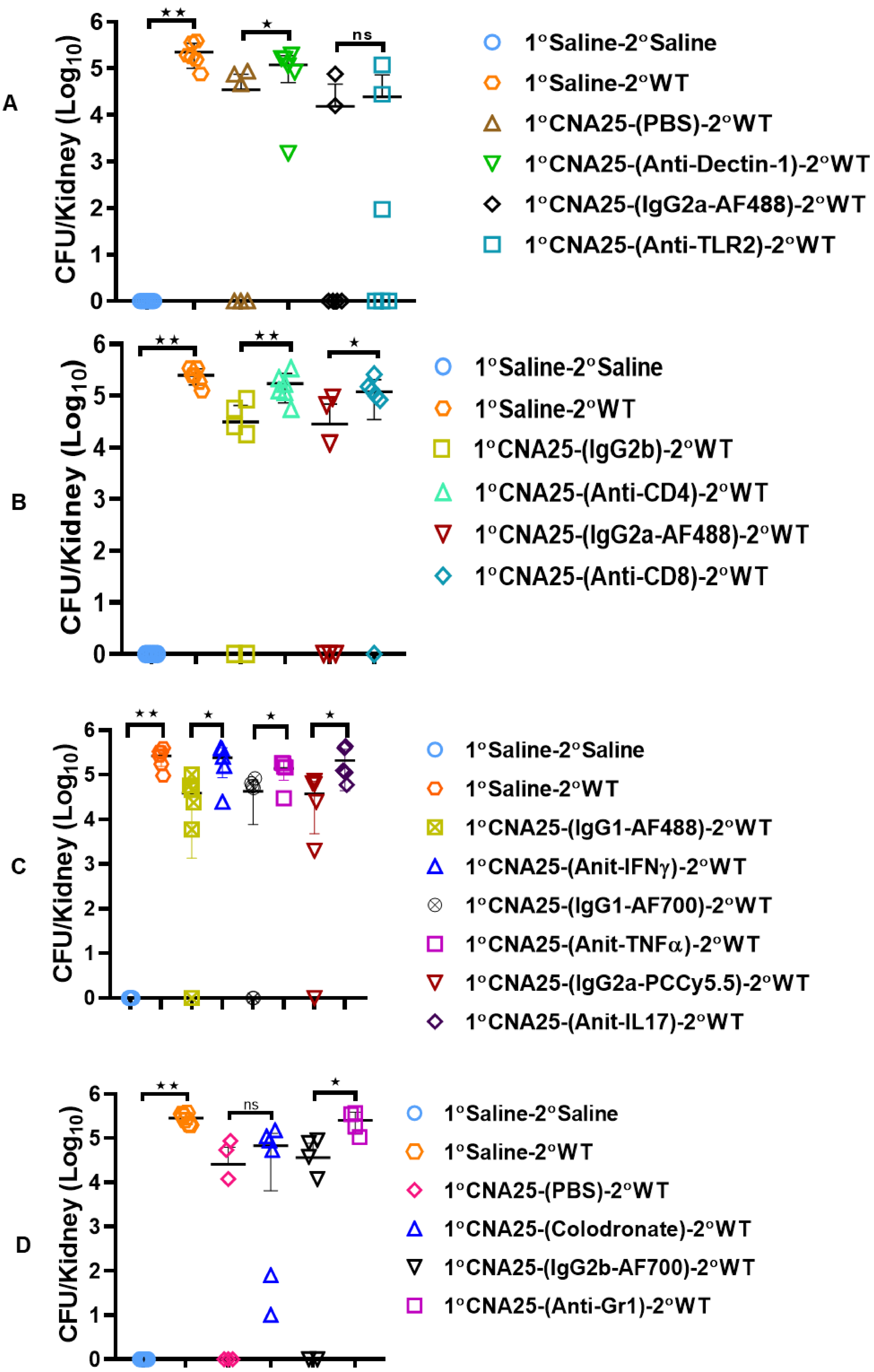

Appendix Figure S4. Fungal burden determined by CFU assay in kidneys of various depleted groups and compared with respective control groups upon saline and WT *C. albicans* lethal challenge. Fungal load is plotted in scattered plots and data were analyzed using the Mann-Whitney U test; **** p ≤ 0.0001; *** p ≤ 0.001; ** p≤ 0.01; * p ≤ 0.05.
