# Supplementary material for: Immunogenicity and efficacy of CNA25 as a potential whole-cell vaccine against systemic candidiasis
Source: EMBO Mol Med. 2024 May 23;16(6):4. doi: 10.1038/s44321-024-00080-8 (PMC11178797; doi:10.1038/s44321-024-00080-8)
Supplement: Supplementary file 5 — Expanded View Figures [file 44321_2024_80_MOESM5_ESM.pdf]

Expanded View Figures

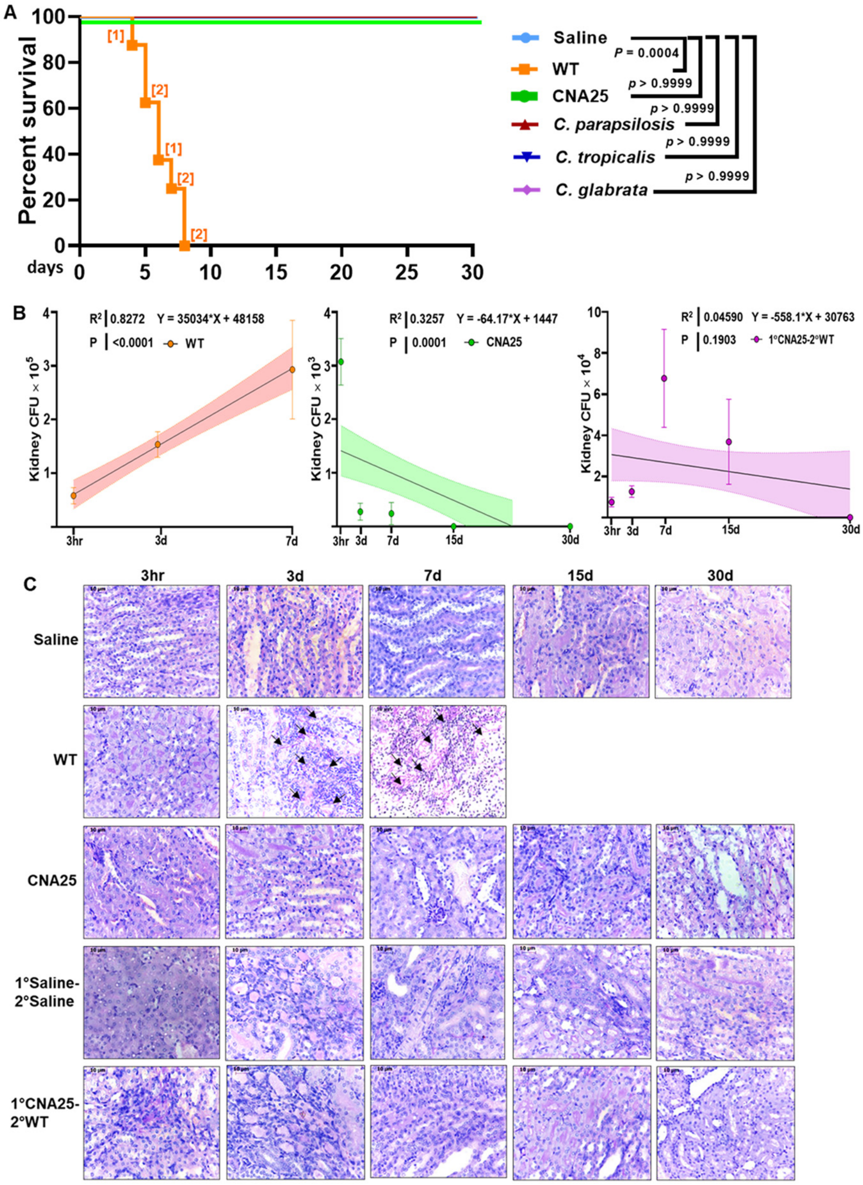

Figure EV1. Virulence of NAC-species and a direct link between disease severity with fungal burden.

(**A**) A Kaplan–Meier survival analysis of BALB/c mice (n = 8) upon intravenous administration of 5 × 10^5 CFU/mice of WT *C. albicans*, CNA25, *C. parapsilosis*, *C. tropicalis*, *C. glabrata*, and saline control (100 µl 1× PBS) for 30 days was conducted. Statistical significance among the comparing groups was determined using the log-rank (Mantel–Cox) test and the *P* values are listed as per the comparisons. (**B**) A simple linear regression analysis of kidney fungal burden in WT, CNA25, and 1°CNA25-2°WT with time (3 h, 3d, 7d, 15d, and 30d) and respective R^2, Equation and p values are given. (**C**) A time kinetic representative kidney PAS staining images of each group (Saline, WT, CNA25, 1°Saline-2°Saline, and 1°CNA25-2°WT) depicting fungal load post-inoculation are shown. Images were acquired in a Leica- ICC50 microscope at × 40 with a 10 µm scale bar. The black arrows indicate hyphal fungal cells.

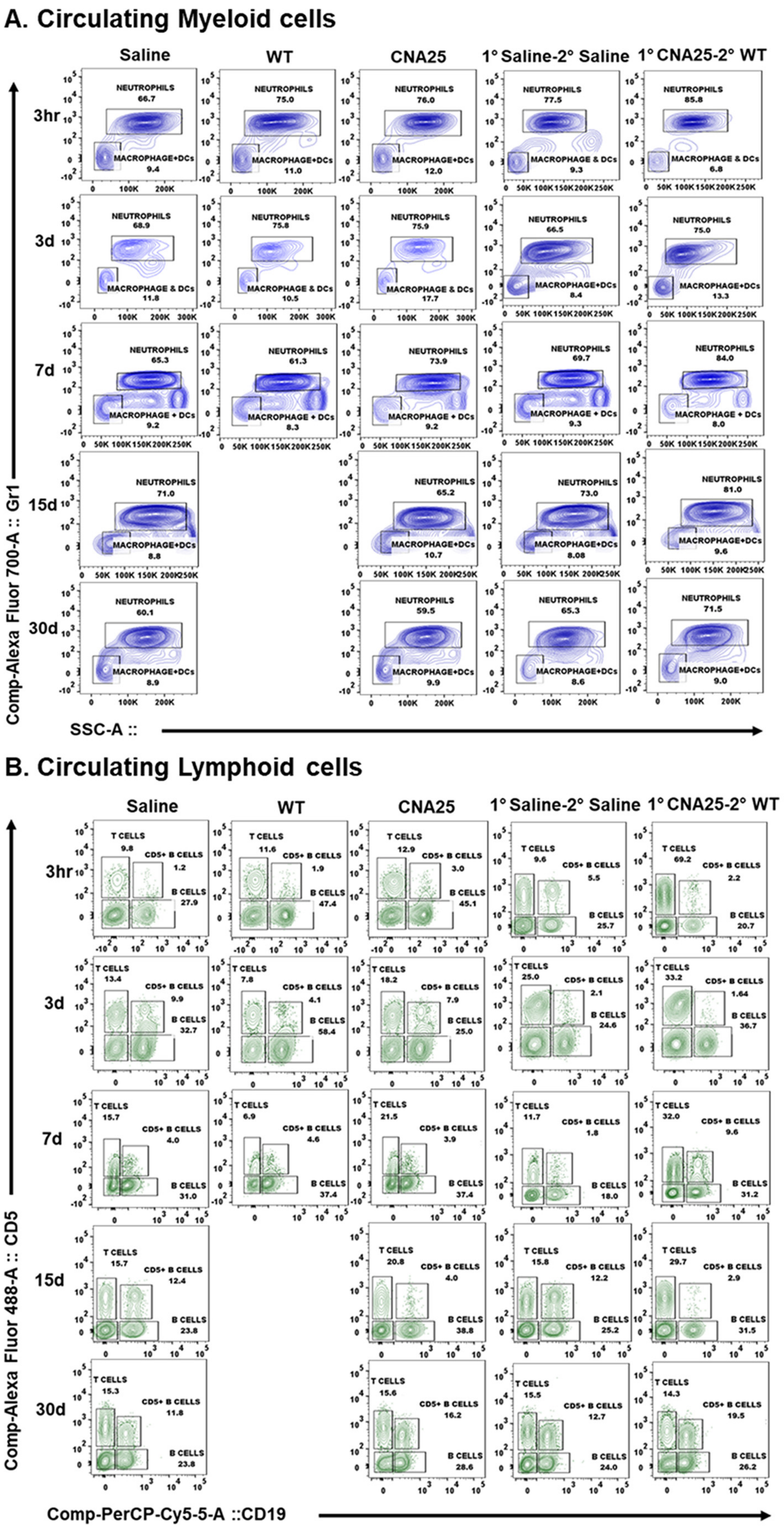
A. Circulating Myeloid cells
Saline
WT
CNA25
1° Saline-2° Saline
1° CNA25-2° WT
3hr
3d
7d
15d
30d
NEUTROPHILS
MACROPHAGE+DCs
Comp-Alexa Fluor 700-A :: Gr1
SSC-A ::
B. Circulating Lymphoid cells
Saline
WT
CNA25
1° Saline-2° Saline
1° CNA25-2° WT
3hr
3d
7d
15d
30d
T CELLS
CD5+ B CELLS
B CELLS
Comp-Alexa Fluor 488-A :: CD5
Comp-PerCP-Cy5-5-A ::CD19

 © The Author(s)

Figure EV2. Circulating myleoid and lymphoid immune cells profiling in various fungal-challenged mice groups.

(**A**) Representative bivariate contour plots of all time points (3 h, 3d, 7d, 15d, and 30d) having Alexa Fluor 700 conjugated Gr-1 Ly6G/Ly6C on *y* axis and Side scatter on *x* axis for the analysis of compartmental distribution of circulating myeloid-derived Macrophage + DCs and neutrophils cells using Flowjo v8.0.2 Software are provided. (**B**) Similar bivariate contour plots of all time points having Alexa Fluor 488 conjugated CD5 on y axis and PerCP-Cy5.5 conjugated CD19 on *x* axis for the analysis of the compartmental distribution of circulating lymphoid-derived B, B1, and T cells using Flowjo v8.0.2 Software are given.

© The Author(s)

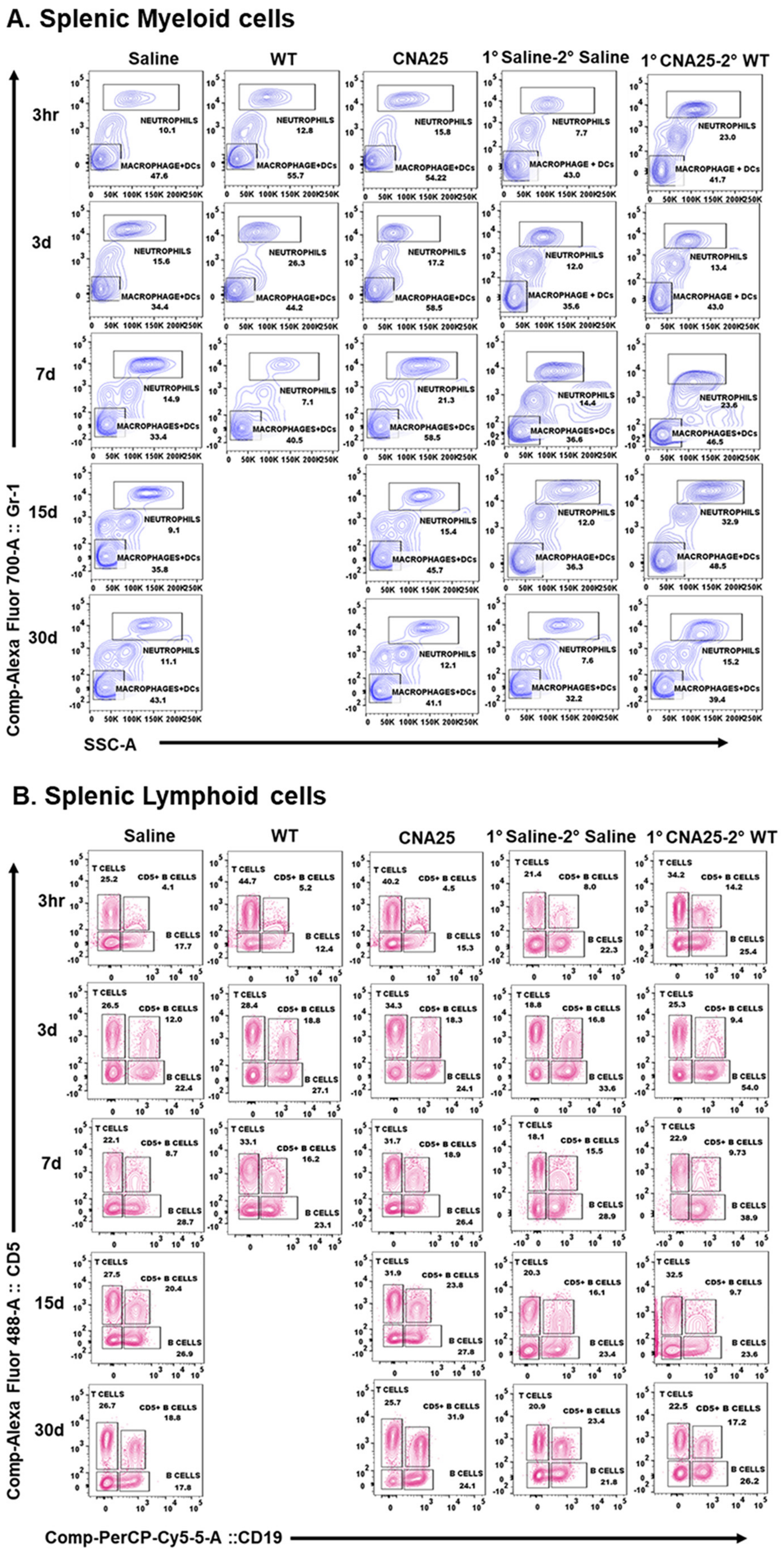
A. Splenic Myeloid cells
Saline
WT
CNA25
1° Saline-2° Saline
1° CNA25-2° WT
3hr
3d
7d
15d
30d
Comp-Alexa Fluor 700-A :: Gr-1
SSC-A
B. Splenic Lymphoid cells
Saline
WT
CNA25
1° Saline-2° Saline
1° CNA25-2° WT
3hr
3d
7d
15d
30d
Comp-Alexa Fluor 488-A :: CD5
Comp-PerCP-Cy5-5-A ::CD19

 © The Author(s)

Figure EV3. Tissue-resident myleoid and lymphoid immune cells profiling in various fungal-challenged mice groups.

(**A**) Representative time kinetic bivariate contour plots having Alexa Fluor 700 conjugated Gr-1 Ly6G/Ly6C on the y axis and Side scatter on the x axis for the analysis of compartmental distribution of splenic myeloid-derived Macrophage + DCs and neutrophils cells using Flowjo v8.0.2 Software are provided. (**B**) Similar bivariate contour plots of each time point having Alexa Fluor 488 conjugated CD5 on the *y* axis and PerCP-Cy5.5 conjugated CD19 on the *x* axis for the analysis of compartmental distribution of splenic lymphoid-derived B, B1, and T cells using Flowjo v8.0.2 Software are shown.

© The Author(s)

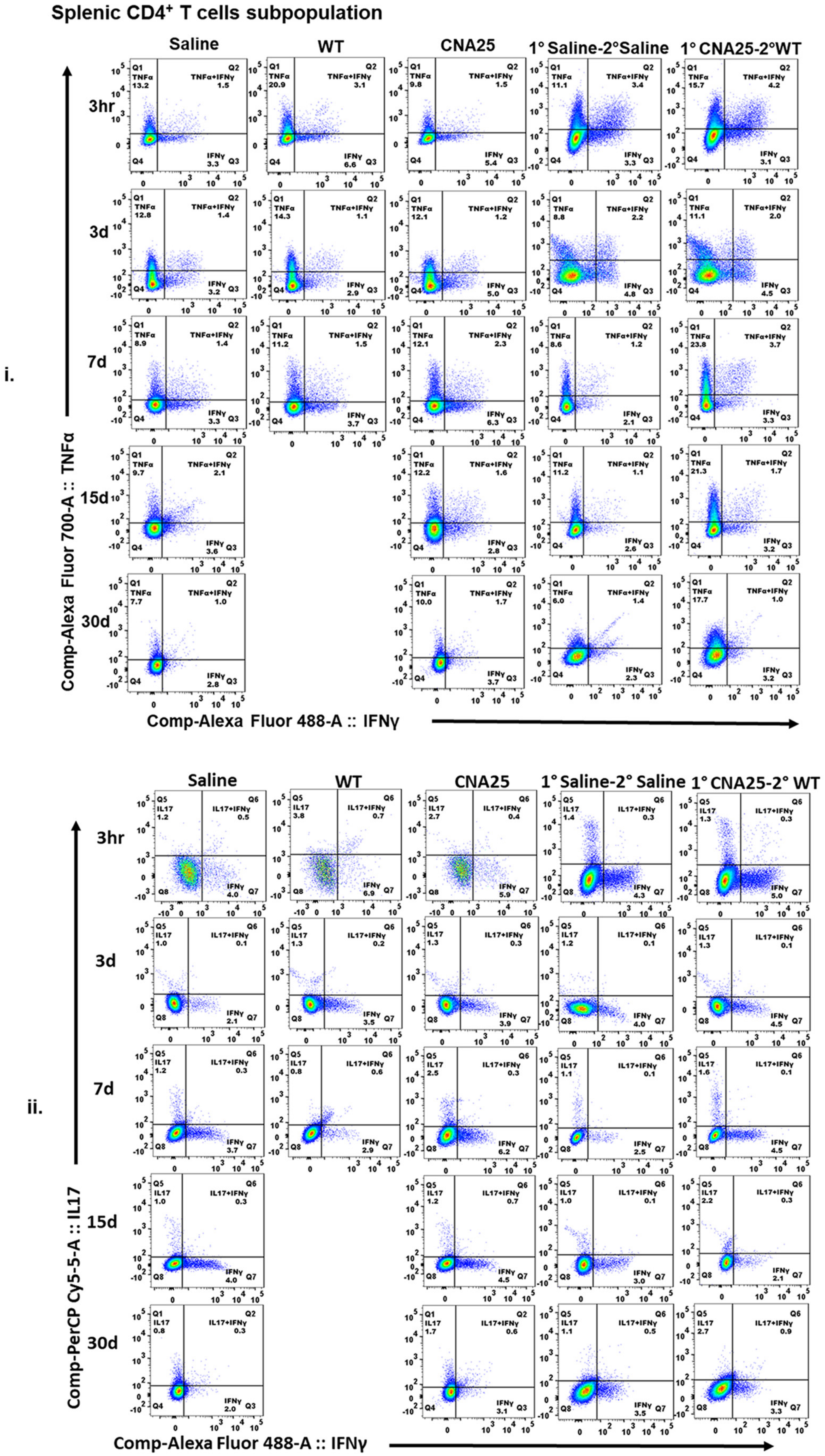
Splenic CD4⁺ T cells subpopulation
i.
Saline
WT
CNA25
1° Saline-2°Saline
1° CNA25-2°WT
3hr
3d
7d
15d
30d
Comp-Alexa Fluor 700-A :: TNFα
Comp-Alexa Fluor 488-A :: IFNγ
ii.
Saline
WT
CNA25
1° Saline-2° Saline
1° CNA25-2° WT
3hr
3d
7d
15d
30d
Comp-PerCP Cy5-5-A :: IL17
Comp-Alexa Fluor 488-A :: IFNγ

 © The Author(s)

Figure EV4. CD4+ T cells profiling based on cytokine in various fungal-challenged mice groups.

(**i**) Representative bivariate pseudo plots of all time points having Alexa Fluor 700 conjugated TNFα on the *y* axis and Alexa fluor 488 conjugated IFNγ on the *x* axis for the analysis of compartmental distribution of splenic $CD4^+$ T cells subpopulation ($IFNγ^+$ $CD4^+$ T cells, $TNFα^+$ $CD4^+$ T cells, and $IFNγ^+TNFα^+$ $CD4^+$ T cells) using Flowjo v8.0.2 Software are given. (**ii**) Representative bivariate pseudo plots of all time points having PerCP-Cy5.5 conjugated IL-17 on the *y* axis and Alexa fluor 488 conjugated IFNγ on the x axis for the analysis of the compartmental distribution of splenic $CD4^+$T cells subpopulation ($IL\text{-}17^+$ $CD4^+$ T cells, $IFNγ^+$ $CD4^+$ T cells, and $IL\text{-}17^+IFNγ^+$ $CD4^+$ T cells) using Flowjo v8.0.2 Software are shown.

© The Author(s)

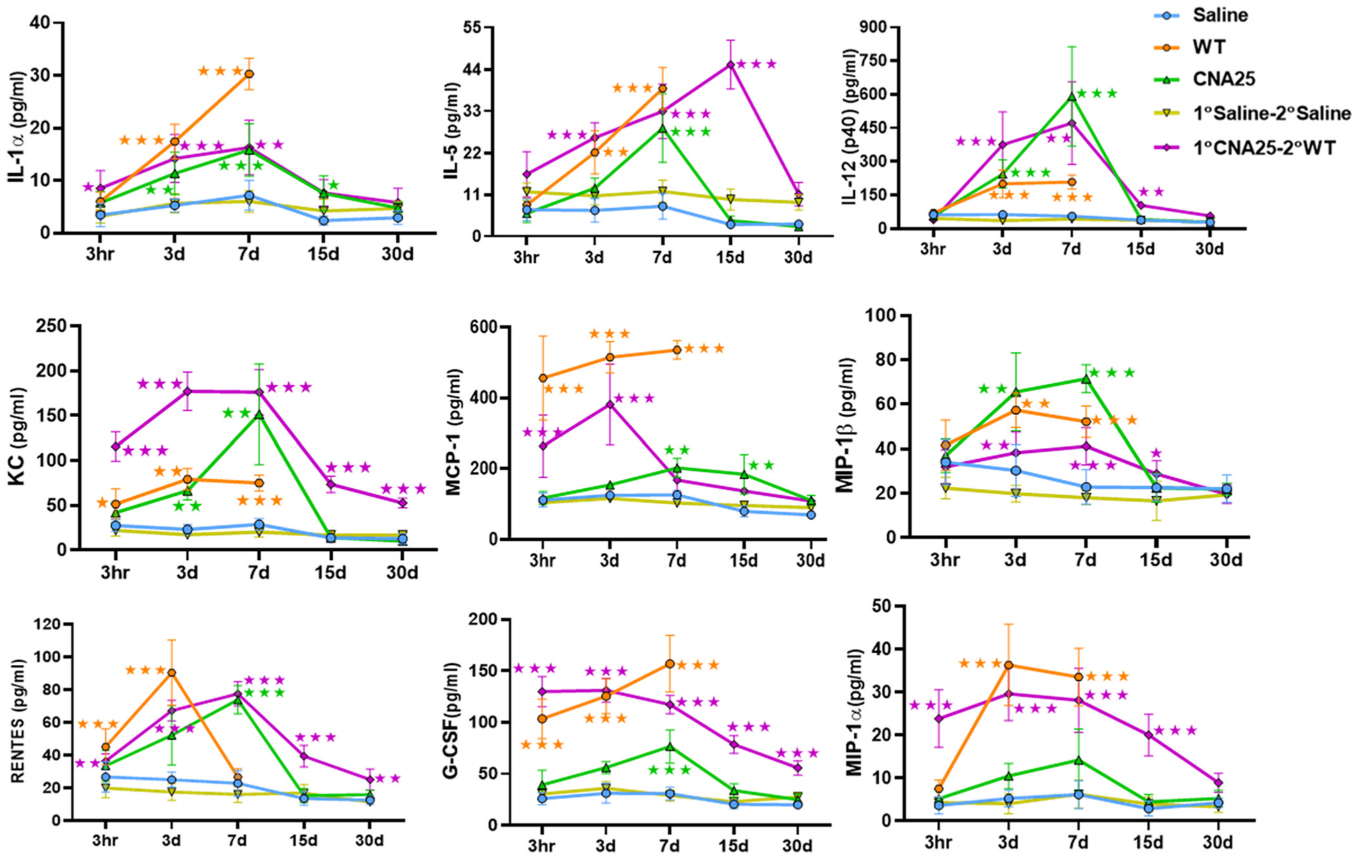

Figure EV5. Serum cytokine profile of infected mice.

The line graphs depicting the concentration of various cytokines and chemokines such as IL-1α, IL-5, IL-12(p40), KC, MCP-1, MIP-1β, MIP-1α, G-CSF, and RENTES in picogram/ml of each mouse of infected groups (WT—Orange, CNA25—green, Saline—sky blue, 1°Saline-2°Saline—greenish yellow, and 1°CNA25-2°WT—purple) at mentioned time points. The lines join the mean± SEM of eight mice data for each group and time point. Data are the representative of two separate experiments and were analyzed using the two-way ANOVA test (Tukey's multiple comparisons test). **P* ≤ 0.05, ***P* ≤ 0.01, ****P* ≤ 0.001, and no star symbols suggest nonsignificant. 1° is primary and 2° denotes re-challenge.

© The Author(s)
